# Sex differences in clinical phenotypes and prognostic associations in acute heart failure

**DOI:** 10.3389/fcvm.2026.2011890

**Published:** 2026-10-28

**Authors:** Georgios Aletras, Maria Bachlitzanaki, Stylianos Fiflis, Eirini Bachlitzanaki, Eleni Eleftheriadou, Yannis Pantazis, Emmanuel Foukarakis, Michalis Hamilos, Konstantinos Stylianou

**Affiliations:** 1 Department of Cardiology, Venizelio General Hospital of Heraklion, Heraklion, Greece; 2 School of Medicine, University of Crete, Heraklion, Greece; 3 Second Department of Internal Medicine, Venizelio General Hospital of Heraklion, Heraklion, Greece; 4 Institute of Applied and Computational Mathematics, Foundation for Research and Technology-Hellas, Heraklion, Greece; 5 Department of Cardiology, University General Hospital of Heraklion, Heraklion, Greece; 6 Department of Nephrology, University General Hospital of Heraklion, Heraklion, Greece

**Keywords:** acute heart failure, cardiorenal, ferritin, inflammation, metabolic markers, prognosis, sex differences, uric acid

## Abstract

**Background:**

Women are under-represented in heart failure trials, and the risk markers used in acute heart failure (AHF) derive largely from male-predominant cohorts. In this study, we compared the metabolic, inflammatory, and renal profiles of men and women hospitalized for AHF and tested whether the prognostic value of these markers differs by sex.

**Methods:**

We analyzed 530 consecutive patients from a prospective single-center AHF registry. Postdischarge mortality was modeled by Cox regression in the 503 patients discharged alive; each of eight covariates was tested for a sex interaction, and 19 metabolic, inflammatory, and renal markers were screened for effect modification by sex.

**Results:**

The total number of male patients was 284 (53.6%). Women were older (82 vs. 79 years) than men and more often had preserved ejection fraction (65.9% vs. 35.9%), frailty (74.0% vs. 59.9%), and chronic kidney disease; they had a higher total cholesterol level (139 vs. 126 mg/dL), a lower troponin level, a higher E/e′ ratio within preserved ejection fraction, and a lower ferritin level (42 vs. 71 ng/mL; below 100 ng/mL in 76.8% vs. 63.7%). Men more often had an ischemic etiology (45.8% vs. 15.0%), although coronary anatomy was more often unassessed in women (all *p* < 0.05). In-hospital (5.3% vs. 4.9%) and postdischarge mortality rates (23.6% vs. 26.7%) and renal outcomes did not differ; after full adjustment, male sex was associated with higher postdischarge mortality [hazard ratio (HR) 1.51, 95% confidence interval (CI) 1.03–2.22], but this depended on the inclusion of the left ventricular ejection fraction (LVEF) category. No covariate showed significant effect modification by sex (all *p* for interaction ≥0.06), with similar optimism-corrected C-indices (men 0.683, women 0.687). Among the 19 markers, only uric acid showed an interaction (women HR 1.15 per mg/dL, 95% CI 1.00–1.33; men 0.91, 0.80–1.03; *p* = 0.008), not surviving multiplicity correction.

**Conclusions:**

Men and women hospitalized for AHF are clinically and biochemically distinct populations. Unadjusted mortality rates were similar in both sexes; an adjusted association with male sex was sensitive to the inclusion of the LVEF category and therefore should be interpreted cautiously. No robust sex-related effect modification was found; given the limited number of events, this represents the absence of evidence rather than evidence of similar prognostic associations between the sexes. Low ferritin levels, frequent in women, warrants assessment with complete iron indices; the uric acid interaction is hypothesis-generating.

## 1 Introduction

Women account for roughly half of patients hospitalized for acute heart failure (AHF) but have been consistently under-represented in randomized trials, and the prognostic models in routine use were derived predominantly from male cohorts (1, 2). Sex differences in the clinical presentation of heart failure (HF) are well described: women are older at presentation, more often have preserved ejection fraction (EF), and carry a different burden of comorbidity (1, 3, 4). Whether these differences extend to the prognostic behavior of the markers used at the bedside is much less clear.

This factor matters practically. If a marker such as natriuretic peptide, uric acid, or an inflammatory index carries a different hazard in women than in men, then a single risk model applied to both sexes will misclassify one of them. If instead the markers behave similarly, the difference between men and women lies in what they present with rather than in how their risk should be read.

Metabolic and inflammatory pathways are a plausible place for such differences to arise. Lipid handling, iron stores, urate metabolism, and the inflammatory response all differ between the sexes, and several of these are altered further by HF itself. Yet, studies that compare men and women with AHF have generally reported differences in the distribution of these markers rather than in their association with outcome.

In a prospective single-center registry of consecutive patients hospitalized for AHF, we therefore set out to (i) describe the metabolic, inflammatory, and renal profiles of men and women; (ii) compare their in-hospital, postdischarge, and renal outcomes; and (iii) test whether the prognostic value of routinely measured markers is modified by sex.

## 2 Materials and methods

### 2.1 Study design and population

This was a single-center observational study of a prospective registry of consecutive adults admitted with a primary diagnosis of AHF to the Cardiology Department of Venizelio General Hospital, Heraklion, Crete, Greece, between February 2023 and June 2025, with follow-up completed in June 2026. The diagnosis of AHF was made according to the 2021 European Society of Cardiology (ESC) guidelines on the basis of symptoms and signs of congestion supported by natriuretic peptide elevation and echocardiographic findings (5, 6). Patients younger than 18 years, those already receiving renal replacement therapy (RRT), and those admitted primarily for an acute coronary syndrome without a dominant AHF presentation were not eligible for the study, nor were admissions in which HF was not the principal reason for admission; only the first eligible admission per patient was included. Of 592 patients assessed for eligibility, 62 met one or more exclusion criteria, leaving a final analysis cohort of 530 patients; none was lost to follow-up. Data from this registry have been reported previously in an analysis of period-specific mortality and of the prediction of postdischarge events according to left ventricular ejection fraction (LVEF) phenotype (7) and in an analysis of multivalvular involvement and 1-year outcomes (8). Neither analysis examined sex differences. The present analysis addresses the distinct question of sex-related differences in clinical and biochemical phenotypes and of potential effect modification of prognostic associations by sex, and the overlap in the cohort and follow-up is declared here for the sake of transparency.

The study was approved by the Institutional Ethics Committee of Venizelio General Hospital of Heraklion (protocol 15/05-08-2022) and conducted in accordance with the Declaration of Helsinki. All participants provided written informed consent.

### 2.2 Data collection and definitions

Demographic data, comorbidities, clinical presentation, and laboratory results were recorded prospectively at admission, and echocardiographic measurements were taken during the index hospitalization period. Sex was recorded as documented in the medical record; no data on gender identity were collected, and the term “sex” is used throughout.

LVEF was measured by the biplane Simpson method and categorized as preserved (LVEF ≥50%) or LVEF <50% using the acute-setting threshold of the 2021 ESC guidelines (5). A single transthoracic echocardiogram was performed in every patient during the index admission period, on median hospital day 3 (interquartile range 2–5; range 1–17). In addition to LVEF, pulmonary artery systolic pressure (PASP), the ratio of early mitral inflow velocity to early diastolic mitral annular velocity (E/e′), left atrial volume index (LAVi), tricuspid annular plane systolic excursion (TAPSE), tricuspid annular systolic velocity (RV S’), left ventricular (LV) and right ventricular (RV) dilation, defined on two-dimensional imaging by the sex-specific reference limits of the American Society of Echocardiography/European Association of Cardiovascular Imaging (ASE/EACVI) chamber quantification recommendations (9), and the echocardiographic probability of pulmonary hypertension (PH), graded as low, intermediate, or high according to the 2022 ESC/European Respiratory Society (ERS) guidelines (10), were recorded, and each valve lesion was graded as none or mild, moderate, or severe. Consistent with the Universal Definition of Heart Failure (6) and with previous analyses of this registry (7), an EF below 50% was analyzed as a single category; because it combines mildly reduced and reduced EF, it is designated throughout as LVEF <50% and is not called “reduced.” This dichotomy has since been adopted by the 2026 ESC guidelines for the management of heart failure, which eliminated the mildly reduced phenotype and defined HF with reduced EF as LVEF <50% (11). Frailty was graded on admission using the Clinical Frailty Scale (CFS), with frailty being defined as a score of 5 or more (12). Chronic kidney disease (CKD) was defined as an estimated glomerular filtration rate (eGFR) below 60 mL/min/1.73 m^2^ persisting for at least 3 months and was staged according to the Kidney Disease: Improving Global Outcomes (KDIGO) 2012 classification (13), with the eGFR being calculated with the 2021 race-free Chronic Kidney Disease Epidemiology Collaboration (CKD-EPI) creatinine equation (14). Baseline creatinine and the derived eGFR were obtained from stable outpatient values recorded in the year before admission rather than from the presentation value, which is distorted by acute congestion; where several values were available, the most representative stable value was selected clinically, excluding measurements obtained during intercurrent illness. These definitions are identical to those used in previous analyses of this registry (7). Worsening renal function (WRF) during the index admission was graded as an ordinal class from the ratio of peak to baseline serum creatinine (class 0, <1.25; 1, 1.25–1.50; 2, 1.51–2.00; 3, 2.01–3.00; 4, >3.00; 5, need for RRT), as in previous analyses of this registry (7). Acute kidney injury (AKI) and WRF class describe related but distinct constructs, and therefore, both are presented: AKI is the binary KDIGO definition, reported as an in-hospital outcome, whereas WRF class is an ordinal measure of the magnitude of creatinine rise and is used only as a prognostic covariate; both are referenced to the same stable preadmission baseline creatinine.

Metabolic markers comprised diabetes mellitus, glycated hemoglobin (HbA1c), admission glucose, uric acid, total cholesterol, low-density lipoprotein (LDL) and high-density lipoprotein (HDL) cholesterol, triglycerides, ferritin, thyroid-stimulating hormone (TSH), magnesium, and albumin. Low ferritin was defined as a serum ferritin level below 100 ng/mL, the threshold used for absolute iron deficiency in the HF guidelines. Transferrin saturation was not measured, and therefore, functional iron deficiency could not be identified, and the term ‘low ferritin’ is used throughout in preference to iron deficiency. Inflammatory markers comprised C-reactive protein (CRP), white cell count, the neutrophil-to-lymphocyte, platelet-to-lymphocyte, and monocyte-to-lymphocyte ratios, and red cell distribution width (RDW). Troponin, aminotransferase, gamma-glutamyl transferase (*γ*GT), and alkaline phosphatase (ALP) levels were also recorded. Inflammatory markers, glucose, magnesium, albumin, troponin, and liver tests were measured on the admission sample; the remaining markers, including lipids, HbA1c, TSH, uric acid, and ferritin, were measured on the first morning sample after admission. HF therapy was recorded at admission and at discharge.

### 2.3 Outcomes

Patients were followed up for 12 months from admission. Mortality was ascertained for the entire cohort from hospital records and the national registry. In-hospital death and postdischarge death were treated as separate outcomes. Renal outcomes comprised in-hospital AKI, defined according to KDIGO criteria as an in-hospital rise in serum creatinine of ≥0.3 mg/dL within 48 h or to ≥1.5 times the stable preadmission baseline value (15) and the need for RRT. WRF class was not analyzed as an outcome but was retained as a prognostic covariate in the multivariable model.

### 2.4 Statistical analysis

Continuous variables were assessed for normality and presented as median (interquartile range), compared with the Mann–Whitney U test; categorical variables were presented as *n* (%) and compared with the *χ*^2^ or Fisher's exact test. Echocardiographic variables that differed between the sexes were additionally compared within each LVEF category to separate differences attributable to sex from those attributable to the different distribution of LVEF phenotype. Time-to-event analyses used Kaplan–Meier estimation with the log-rank test.

In-hospital and postdischarge deaths are governed by different processes and cannot be assumed to share a common hazard ratio (HR). Therefore, mortality was analyzed in two periods. In-hospital mortality was described but not modeled, given that there were only 27 events. Postdischarge all-cause mortality, with time zero at discharge, was the sole endpoint for multivariable modeling and was analyzed by Cox proportional-hazards regression in the 503 patients discharged alive. Because follow-up ended 12 months after admission, the postdischarge observation window varied with the length of stay (median 51.0, range 44.7–51.7 weeks); patients were censored at their individual end of follow-up.

Covariates were selected *a priori* on the basis of established prognostic relevance in AHF and constrained by the number of events: sex, age, frailty, log N-terminal pro–B-type natriuretic peptide (NT-proBNP), baseline eGFR, hemoglobin, sodium, WRF class, and LVEF category. No automated variable selection was used. The model was fitted by a complete-case analysis; missing data were confined to NT-proBNP, which was unavailable in nine of the 503 patients discharged alive. The proportional-hazards assumption was examined by testing the correlation between scaled Schoenfeld residuals and rank-transformed time, and the linearity of each continuous covariate by comparing the linear model with a restricted cubic spline (three knots) using a likelihood-ratio test. Where a covariate violated the proportional-hazards assumption, the primary model was refitted with that covariate omitted and, separately, stratified on the tertiles of that covariate, to confirm that the sex coefficient and the interaction conclusions were unaffected. Discrimination was summarized by using Harrell's C-index. Optimism was estimated by bootstrap resampling with 500 replications: in each replication, the model was refitted in the bootstrap sample and evaluated both in that sample and in the original data, and the optimism-corrected C-index is the apparent C-index minus the mean of these differences. The reported 95% confidence interval (CI) is the 2.5th to 97.5th percentile of the optimism-corrected statistic across replications and therefore describes the corrected rather than the apparent estimate.

Effect modification by sex was assessed in two ways. The primary approach was a formal sex-by-covariate interaction term added to the combined model, one covariate at a time, tested by using the Wald statistic; each such model retained all main effects, including sex and the covariate concerned, in addition to the interaction term. All *p*-values for interaction reported for the prognostic model are obtained from these combined-model interaction terms. Sex-stratified models are additionally presented for description; with 71 deaths in men and 53 in women, these stratified estimates are imprecise and are not intended as separate prognostic models. Second, each of 19 metabolic, inflammatory, and renal markers was added in turn to a core model comprising age, frailty, log NT-proBNP, eGFR, and LVEF category, together with both main effects—sex and the marker—and a marker-by-sex interaction term, the interaction being tested by using the Wald statistic. The 19 markers were diabetes mellitus, HbA1c, admission glucose, uric acid, total cholesterol, LDL and HDL cholesterol, triglycerides, ferritin as a continuous variable, low ferritin (<100 ng/mL), albumin, CRP, white cell count, the neutrophil-to-lymphocyte, platelet-to-lymphocyte, and monocyte-to-lymphocyte ratios, RDW, CKD, and WRF class. TSH and magnesium were measured and are described with the other laboratory markers but were not included in the interaction screen. Markers with a wide range were standardized or log-transformed as indicated. These analyses were exploratory and were not adjusted for multiple comparisons; the Bonferroni threshold for 19 tests (*p* < 0.0026) is given for reference. Any marker showing evidence of interaction was examined further in a series of sensitivity analyses.

A two-sided *p* < 0.05 was considered significant. Analyses were performed in Python 3.12 (Python Software Foundation, Wilmington, DE, USA) using lifelines 0.27.8, pandas 2.2.1, NumPy 1.26.4, SciPy 1.12.0, statsmodels 0.14.1, and Matplotlib 3.10.8.

## 3 Results

### 3.1 Clinical profile

Of the 530 patients, 284 (53.6%) were men and 246 (46.4%) women. Women were older [median 82 (76–87) vs. 79 (71–85) years, *p* = 0.004], more often had atrial fibrillation (AF) (64.6% vs. 54.2%, *p* = 0.019), CKD (56.5% vs. 47.5%, *p* = 0.048), and frailty (74.0% vs. 59.9%, *p* = 0.001), with a larger proportion in CFS 6–9 (45.5% vs. 27.8%) and in New York Heart Association (NYHA) class IV (19.5% vs. 12.7%) (both *p* < 0.001), and presented more often with acute pulmonary edema (29.3% vs. 21.5%, *p* = 0.050). Men more frequently had an ischemic etiology (45.8% vs. 15.0%, *p* < 0.001), previous coronary artery bypass grafting (17.6% vs. 4.9%, *p* < 0.001), and more often reported active smoking (15.1% vs. 5.7%, *p* = 0.001) and airway disease (41.2% vs. 21.1%, *p* < 0.001). Coronary artery disease was documented in 59.5% of men and 28.0% of women, but coronary anatomy had not been assessed in 45.5% of women compared with 22.5% of men (*p* < 0.001 across categories). Previous HF hospitalization, right HF, and the mode of presentation otherwise did not differ (Table 1).

**Table 1:** Baseline clinical characteristics by sex.

Characteristic	All (*n* = 530)	Men (*n* = 284)	Women (*n* = 246)	*p*
Demographics and heart failure profile				
Age, years	81 [74–86]	79 [71–85]	82 [76–87]	0.004
*De novo* heart failure	146 (27.5)	79 (27.8)	67 (27.2)	0.959
Previous HF hospitalization (last 2 years)	142 (26.8)	70 (24.6)	72 (29.3)	0.272
Frailty (CFS ≥ 5)	352 (66.4)	170 (59.9)	182 (74.0)	0.001
CFS category				<0.001
CFS 1–3	44 (8.3)	24 (8.5)	20 (8.1)	
CFS 4–5	295 (55.7)	181 (63.7)	114 (46.3)	
CFS 6–9	191 (36.0)	79 (27.8)	112 (45.5)	
Baseline NYHA class				<0.001
I	22 (4.2)	11 (3.9)	11 (4.5)	
II	103 (19.4)	76 (26.8)	27 (11.0)	
III	321 (60.6)	161 (56.7)	160 (65.0)	
IV	84 (15.8)	36 (12.7)	48 (19.5)	
Right heart failure	226 (42.6)	121 (42.6)	105 (42.7)	1.000
Comorbidities				
Ischemic etiology	167 (31.5)	130 (45.8)	37 (15.0)	<0.001
Coronary artery disease				<0.001
Documented	238 (44.9)	169 (59.5)	69 (28.0)	
Absent	116 (21.9)	51 (18.0)	65 (26.4)	
Not assessed	176 (33.2)	64 (22.5)	112 (45.5)	
Previous CABG	62 (11.7)	50 (17.6)	12 (4.9)	<0.001
Atrial fibrillation	313 (59.1)	154 (54.2)	159 (64.6)	0.019
Type of atrial fibrillation (*n* = 313)				0.954
Paroxysmal	101 (32.3)	50 (32.5)	51 (32.1)	
Persistent or permanent	184 (58.8)	91 (59.1)	93 (58.5)	
Unknown	28 (8.9)	13 (8.4)	15 (9.4)	
Arterial hypertension	462 (87.2)	241 (84.9)	221 (89.8)	0.114
Diabetes mellitus	275 (51.9)	159 (56.0)	116 (47.2)	0.052
Chronic kidney disease	274 (51.7)	135 (47.5)	139 (56.5)	0.048
Airway disease	169 (31.9)	117 (41.2)	52 (21.1)	<0.001
Prior stroke or TIA	39 (7.4)	21 (7.4)	18 (7.3)	1.000
Current smoking	57 (10.8)	43 (15.1)	14 (5.7)	0.001
Active malignancy	51 (9.6)	32 (11.3)	19 (7.7)	0.218
Previous malignancy	36 (6.8)	16 (5.6)	20 (8.1)	0.334
Implantable defibrillator	31 (5.8)	29 (10.2)	2 (0.8)	<0.001
Permanent pacemaker	51 (9.6)	26 (9.2)	25 (10.2)	0.807
Clinical presentation				
Systolic BP on admission, mmHg	145 [127–170]	142 [120–169]	147 [130–170]	0.084
Acute decompensated heart failure	389 (73.4)	217 (76.4)	172 (69.9)	0.112
Acute pulmonary edema	133 (25.1)	61 (21.5)	72 (29.3)	0.050
Cardiogenic shock	6 (1.1)	5 (1.8)	1 (0.4)	0.223
Isolated right heart failure	2 (0.4)	1 (0.4)	1 (0.4)	1.000

BP, blood pressure; CABG, coronary artery bypass grafting; CFS, clinical frailty scale; HF, heart failure; NYHA, New York Heart Association; TIA, transient ischemic attack.

Continuous variables are median [interquartile range]; categorical variables are *n* (%). Ordinal variables (CFS category and NYHA class) were compared with the Mann–Whitney U test. Coronary artery disease was classified as documented, absent, or not assessed, the last denoting patients in whom coronary anatomy had not been evaluated. The type of atrial fibrillation is shown among the 313 patients with atrial fibrillation.

Preserved EF was present in 65.9% of women and 35.9% of men (*p* < 0.001). On echocardiography, men more frequently had LV dilation (34.9% vs. 14.6%, *p* < 0.001), a difference that persisted within the LVEF <50% group (52.2% vs. 33.3%, *p* = 0.006). Women had a higher E/e′ ratio [18.0 (14.0–24.4) vs. 16.0 ([12.5–22.0), *p* = 0.003] and a higher estimated PASP [45 (35–55) vs. 40 (30–55) mmHg, *p* = 0.024], and a high echocardiographic probability of PH was slightly more frequent in women (57.7% vs. 48.9%; *p* = 0.050 across categories). The difference in E/e′ was confined to patients with LVEF ≥50%, in whom it was higher in women than in men [18.0 (14.0–24.0) vs. 14.0 (11.0–17.9), *p* < 0.001], whereas it did not differ in those with LVEF <50% (*p* = 0.164); PASP, by contrast, did not differ between the sexes within either LVEF group (median 45 vs. 45 mmHg with LVEF ≥50%, *p* = 0.70; 40 vs. 40 mmHg with LVEF <50%, *p* = 0.14), indicating that the overall difference reflected the larger proportion of women with preserved EF, in whom PASP was higher. LAVi, RV function (TAPSE and RV S’), and RV dilation were similar in men and women. Among valve lesions, moderate or severe tricuspid regurgitation (TR) (52.8% vs. 35.9%, *p* < 0.001) and mitral stenosis (7.7% vs. 1.1%, *p* < 0.001) were more frequent in women, whereas mitral regurgitation, aortic stenosis, aortic regurgitation, and previous valve replacement did not differ between the sexes. HF therapy at admission differed little: women more often received an angiotensin-converting enzyme inhibitor (ACEi) or angiotensin receptor blocker (ARB) (57.3% vs. 44.4%, *p* = 0.004) and less often an angiotensin receptor–neprilysin inhibitor (ARNI) (1.6% vs. 5.6%, *p* = 0.029). At discharge, women were less often prescribed a sodium–glucose cotransporter-2 inhibitor (SGLT2i) (58.4% vs. 69.3%, *p* = 0.014) or a statin (60.1% vs. 70.7%, *p* = 0.016), whereas beta-blocker, mineralocorticoid receptor antagonist (MRA), and renin–angiotensin system (RAS) inhibitor prescriptions did not differ between the sexes (Table 2).

**Table 2:** Echocardiographic findings and heart failure therapy by sex.

Characteristic	All (*n* = 530)	Men (*n* = 284)	Women (*n* = 246)	*p*
Echocardiography				
LVEF, %	45 [30–55]	35 [25–55]	52 [45–60]	<0.001
LVEF ≥50%	264 (49.8)	102 (35.9)	162 (65.9)	<0.001
PASP, mmHg (*n* = 529)	40 [35–55]	40 [30–55]	45 [35–55]	0.024
E/e′ ratio (*n* = 519)	16.5 [13.0–23.0]	16.0 [12.5–22.0]	18.0 [14.0–24.4]	0.003
LAVi, mL/m^2^ (*n* = 529)	51 [44–63]	51 [44–62]	51 [43–65]	0.737
TAPSE, mm (*n* = 529)	18 [15–19]	18 [15–19]	18 [16–20]	0.976
RV S’, cm/s (*n* = 529)	10 [9–12]	10 [9–12]	10 [9–12]	0.968
LV dilation	135 (25.5)	99 (34.9)	36 (14.6)	<0.001
RV dilation	130 (24.5)	70 (24.6)	60 (24.4)	1.000
Echocardiographic probability of PH				0.050
Low	75 (14.2)	44 (15.5)	31 (12.6)	
Intermediate	174 (32.8)	101 (35.6)	73 (29.7)	
High	281 (53.0)	139 (48.9)	142 (57.7)	
Valvular heart disease (moderate or severe)				
Tricuspid regurgitation	232 (43.8)	102 (35.9)	130 (52.8)	<0.001
Mitral regurgitation	227 (42.8)	120 (42.3)	107 (43.5)	0.841
Mitral stenosis	22 (4.2)	3 (1.1)	19 (7.7)	<0.001
Aortic stenosis	105 (19.8)	57 (20.1)	48 (19.5)	0.959
Aortic regurgitation	67 (12.6)	36 (12.7)	31 (12.6)	1.000
Multivalvular disease	185 (34.9)	89 (31.3)	96 (39.0)	0.078
Previous valve replacement	33 (6.2)	19 (6.7)	14 (5.7)	0.768
Heart failure therapy at admission				
ACEi or ARB	267 (50.4)	126 (44.4)	141 (57.3)	0.004
ARNI	20 (3.8)	16 (5.6)	4 (1.6)	0.029
Beta-blocker	302 (57.0)	163 (57.4)	139 (56.5)	0.906
MRA	114 (21.5)	63 (22.2)	51 (20.7)	0.765
SGLT2i	108 (20.4)	65 (22.9)	43 (17.5)	0.152
Furosemide	265 (50.0)	134 (47.2)	131 (53.3)	0.191
Furosemide dose, mg	10 [0–40]	0 [0–40]	20 [0–40]	0.203
Statin	307 (57.9)	176 (62.0)	131 (53.3)	0.052
Heart failure therapy at discharge (*n* = 503; 270 men, 233 women)				
ACEi or ARB	197 (39.2)	97 (35.9)	100 (42.9)	0.131
ARNI	55 (10.9)	36 (13.3)	19 (8.2)	0.087
Beta-blocker	391 (77.7)	215 (79.6)	176 (75.5)	0.321
MRA	293 (58.3)	157 (58.1)	136 (58.4)	1.000
SGLT2i	323 (64.2)	187 (69.3)	136 (58.4)	0.014
Furosemide	446 (88.7)	232 (85.9)	214 (91.8)	0.051
Furosemide dose, mg	40 [40–80]	40 [40–80]	40 [40–60]	0.382
Statin	331 (65.8)	191 (70.7)	140 (60.1)	0.016

ACEi, angiotensin-converting enzyme inhibitor; ARB, angiotensin receptor blocker; ARNI, angiotensin receptor–neprilysin inhibitor; E/e′, ratio of early mitral inflow velocity to early diastolic mitral annular velocity; LAVi, left atrial volume index; LV, left ventricular; LVEF, left ventricular ejection fraction; MRA, mineralocorticoid receptor antagonist; PASP, pulmonary artery systolic pressure; PH, pulmonary hypertension; RV, right ventricular; RV S’, tricuspid annular systolic velocity; SGLT2i, sodium–glucose cotransporter-2 inhibitor; TAPSE, tricuspid annular plane systolic excursion.

Continuous variables are median [interquartile range]; categorical variables are *n* (%). Valve lesions are moderate or severe, unless stated otherwise; multivalvular disease denotes moderate or severe disease of more than one valve, mixed disease of a single valve not being counted as multivalvular and, for prosthetic valves, only prosthetic dysfunction being counted. The echocardiographic probability of PH was compared as an ordinal variable with the Mann–Whitney U test. Discharge therapy is reported for the 503 patients discharged alive.

### 3.2 Metabolic, inflammatory, and renal profiles

The metabolic profile differed appreciably between the sexes. Women had higher levels of total cholesterol [139 (116–164) vs. 126 (105–158) mg/dL, *p* = 0.002], HDL cholesterol [40 (32–49) vs. 35 (30–43) mg/dL, *p* < 0.001], and LDL cholesterol [78 (60–97) vs. 68 (52–96) mg/dL, *p* = 0.010], and markedly lower ferritin levels [42 (26–93) vs. 71 (36–140) ng/mL, *p* < 0.001], with ferritin below 100 ng/mL in 76.8% of women compared with 63.7% of men (*p* = 0.002). Glycemic indices, uric acid, thyroid function, magnesium, and albumin did not differ between the sexes.

Inflammatory indices were largely similar. The monocyte-to-lymphocyte ratio was lower in women [0.4 (0.3–0.6) vs. 0.5 (0.4–0.8), *p* = 0.005] than in men, but CRP, white cell count, the neutrophil-to-lymphocyte and platelet-to-lymphocyte ratios, and RDW did not differ between them.

Women had a more advanced renal phenotype at baseline than men, with a lower eGFR [55 (40–73) vs. 63 (45–79) mL/min/1.73 m^2^, *p* = 0.003] and a shift toward more advanced KDIGO eGFR categories (*p* = 0.011): 56.5% of women vs. 47.5% of men were in category G3a or worse, and 10.2% vs. 5.3% in G4. The same difference was present on admission [eGFR 54 (36–70) vs. 60 (43–78) mL/min/1.73 m^2^, *p* = 0.003]. Women also had a lower hemoglobin level (11.5 vs. 11.9 g/dL, *p* = 0.002). Albuminuria and NT-proBNP did not differ, whereas admission troponin was lower in women [22.9 (12.0–55.6) vs. 35.6 (16.2–87.1) pg/mL, *p* < 0.001]. Among liver tests, *γ*GT was lower [26 (18–50) vs. 35 (21–59) IU/L, *p* < 0.001] and ALP higher [84 (68–107) vs. 77 (61–99) IU/L, *p* = 0.005] in women, with no difference in aminotransferases (Table 3).

**Table 3:** Metabolic, inflammatory, hepatic, renal, and cardiac biomarker profiles by sex.

Marker	All (*n* = 530)	Men (*n* = 284)	Women (*n* = 246)	*p*
Metabolic				
Diabetes mellitus	275 (51.9)	159 (56.0)	116 (47.2)	0.052
HbA1c, % (*n* = 522)	6.1 [5.6–6.9]	6.1 [5.6–6.9]	6.0 [5.6–6.9]	0.484
Admission glucose, mg/dL	132 [109–180]	132 [108–184]	132 [110–175]	0.790
Uric acid, mg/dL (*n* = 414)	7.5 [6.1–9.1]	7.7 [6.4–9.3]	7.3 [5.8–9.0]	0.115
Total cholesterol, mg/dL (*n* = 527)	134 [110–161]	126 [105–158]	139 [116–164]	0.002
LDL cholesterol, mg/dL (*n* = 528)	73 [55–96]	68 [52–96]	78 [60–97]	0.010
HDL cholesterol, mg/dL (*n* = 527)	37 [30–47]	35 [30–43]	40 [32–49]	<0.001
Triglycerides, mg/dL (*n* = 528)	101 [77–130]	101 [76–123]	101 [79–135]	0.134
Ferritin, ng/mL (*n* = 507)	55 [30–118]	71 [36–140]	42 [26–93]	<0.001
Ferritin <100 ng/mL (*n* = 507)	354 (69.8)	172 (63.7)	182 (76.8)	0.002
TSH, mIU/L (*n* = 518)	1.22 [0.72–2.09]	1.20 [0.72–1.95]	1.25 [0.72–2.16]	0.551
Magnesium, mg/dL (*n* = 517)	2.0 [1.8–2.2]	2.0 [1.8–2.2]	2.0 [1.8–2.2]	0.384
Albumin, g/dL (*n* = 524)	4.0 [3.8–4.3]	4.0 [3.8–4.3]	4.0 [3.8–4.3]	0.990
Inflammatory				
C-reactive protein, mg/dL	0.90 [0.40–2.20]	1.00 [0.40–2.50]	0.80 [0.40–2.20]	0.278
White cell count, ×10^9^/L	8.65 [6.80–11.07]	8.33 [6.80–11.03]	8.90 [6.80–11.07]	0.341
Neutrophil-to-lymphocyte ratio	4.2 [2.9–6.4]	4.3 [2.9–6.6]	4.1 [2.9–6.4]	0.525
Platelet-to-lymphocyte ratio	164 [116–240]	157 [116–233]	172 [116–249]	0.239
Monocyte-to-lymphocyte ratio	0.5 [0.3–0.7]	0.5 [0.4–0.8]	0.4 [0.3–0.6]	0.005
Red cell distribution width, %	16.2 [15.0–18.3]	16.1 [14.8–18.2]	16.3 [15.2–18.4]	0.289
Liver function				
AST, IU/L	27 [21–39]	26 [21–37]	29 [21–41]	0.341
ALT, IU/L	19 [13–32]	19 [14–31]	19 [12–32]	0.261
*γ*GT, IU/L (*n* = 516)	30 [20–54]	35 [21–59]	26 [18–50]	<0.001
ALP, IU/L (*n* = 512)	80 [64–103]	77 [61–99]	84 [68–107]	0.005
Renal, hematological, and cardiac				
Admission creatinine, mg/dL	1.14 [0.92–1.53]	1.22 [0.99–1.63]	1.05 [0.85–1.45]	<0.001
Baseline creatinine, mg/dL	1.10 [0.90–1.40]	1.20 [1.00–1.60]	1.00 [0.80–1.30]	<0.001
Admission eGFR, mL/min/1.73 m^2^	57 [40–75]	60 [43–78]	54 [36–70]	0.003
Baseline eGFR, mL/min/1.73 m^2^	58 [43–77]	63 [45–79]	55 [40–73]	0.003
KDIGO GFR category at baseline, mL/min/1.73 m^2^, *n* (%)				0.011
G1 (≥90)	48 (9.1)	32 (11.3)	16 (6.5)	
G2 (60–89)	208 (39.2)	117 (41.2)	91 (37.0)	
G3a (45–59)	125 (23.6)	64 (22.5)	61 (24.8)	
G3b (30–44)	109 (20.6)	56 (19.7)	53 (21.5)	
G4 (15–29)	40 (7.5)	15 (5.3)	25 (10.2)	
UACR, mg/g (*n* = 311)	96 [30–303]	94 [30–283]	98 [29–319]	0.899
Admission sodium, mEq/L	139 [137–141]	139 [137–141]	139 [137–141]	0.908
Hemoglobin, g/dL	11.7 [10.4–13.0]	11.9 [10.5–13.3]	11.5 [10.2–12.7]	0.002
NT-proBNP, pg/mL (*n* = 519)	5,248 [3,032–10,533]	5,488 [3,180–11,345]	5,026 [2,894–10,418]	0.557
Troponin, pg/mL	28.8 [14.3–69.4]	35.6 [16.2–87.1]	22.9 [12.0–55.6]	<0.001

ALP, alkaline phosphatase; ALT, alanine aminotransferase; AST, aspartate aminotransferase; eGFR, estimated glomerular filtration rate; *γ*GT, gamma-glutamyl transferase; HbA1c, glycated hemoglobin; NT-proBNP, N-terminal pro–B-type natriuretic peptide; TSH, thyroid-stimulating hormone; UACR, urinary albumin-to-creatinine ratio.

Values are median [interquartile range] or *n* (%). Denominators are given where values were not available in all patients. Low ferritin was defined as a serum ferritin level below 100 ng/mL. The KDIGO GFR category was compared as an ordinal variable with the Mann–Whitney U test. Baseline creatinine and the eGFR refer to stable preadmission values; admission values refer to the first measurement on presentation. No patient was in category G5 at baseline.

### 3.3 Outcomes

Twenty-seven patients (5.1%) died in hospital, with no difference between the sexes (men 4.9%, women 5.3%, *p* = 1.00), and the in-hospital cardiovascular death rate was likewise similar (4.2% vs. 3.3%, *p* = 0.72). Among the 503 patients discharged alive, postdischarge all-cause mortality was 26.7% in men and 23.6% in women (*p* = 0.49), and the survival curves did not differ (log-rank *p* = 0.43; crude HR for men 1.15, 95% CI 0.81–1.64) (Figure 1). The rates of mortality at 12 months from admission in the whole cohort were 30.3% and 27.6% (*p* = 0.57), and the mortality rates by 6 months from admission were 18.0% and 17.9% in the whole cohort (*p* = 1.00), corresponding to 13.7% and 13.3% among hospital survivors (*p* = 1.00).

**Figure 1:**
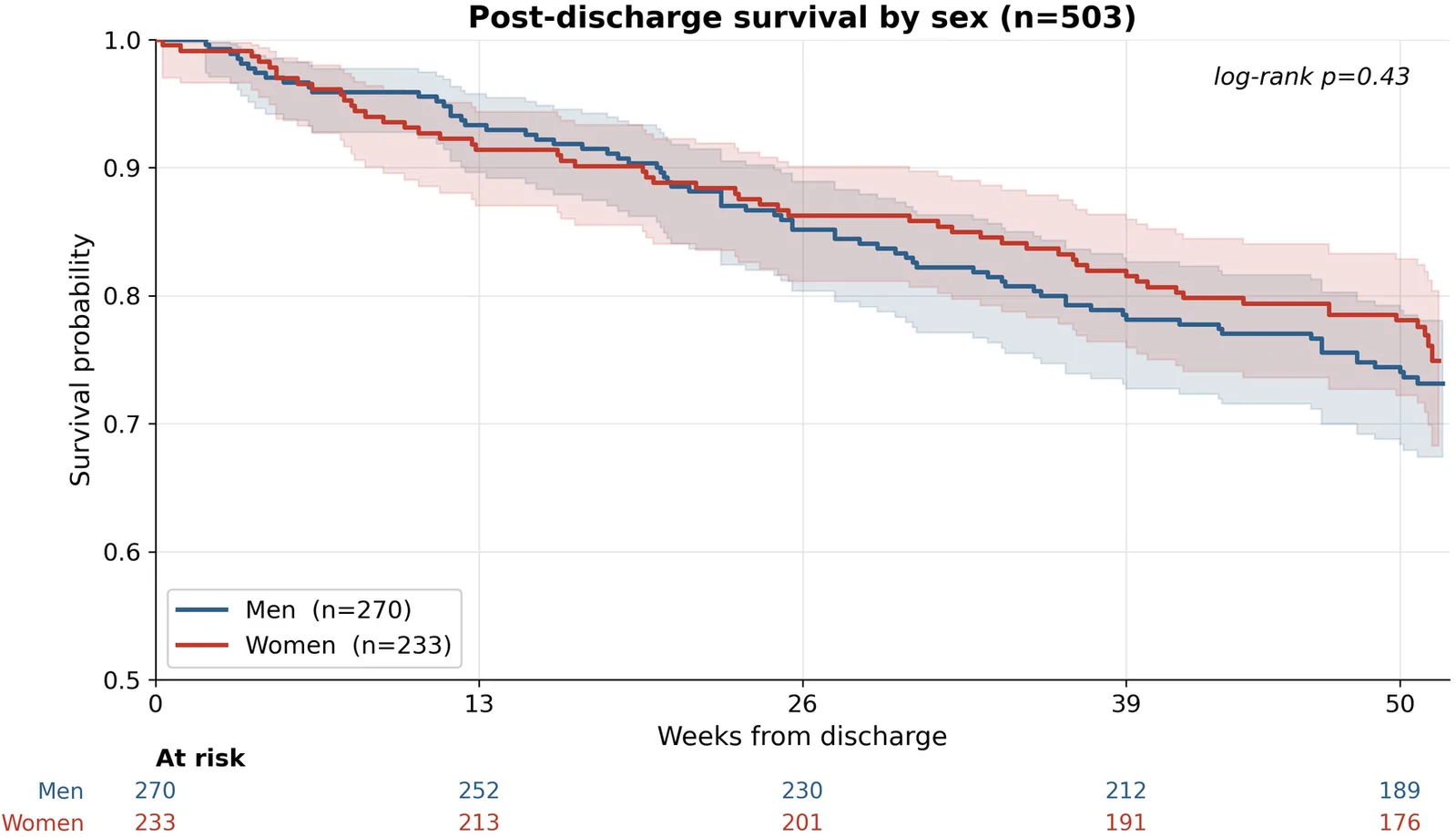
Kaplan–Meier postdischarge all-cause survival by sex in the 503 patients discharged alive, with time zero at discharge. The follow-up ended 12 months after the index admission period, and therefore, the observation window varied with the length of stay. The shaded areas denote 95% confidence intervals; numbers at risk are shown below the axis.

No statistically significant sex differences were observed in the renal outcomes examined. In the whole cohort, in-hospital AKI occurred in 51.1% of men and 44.7% of women (*p* = 0.17) and in-hospital RRT in 2.5% and 2.4% (*p* = 1.00) (Table 4).

**Table 4:** In-hospital course and outcomes by sex.

Outcome	All	Men	Women	*p*
In-hospital course (*n* = 530; 284 men, 246 women)				
In-hospital acute kidney injury (KDIGO)	255 (48.1)	145 (51.1)	110 (44.7)	0.171
In-hospital renal replacement therapy	13 (2.5)	7 (2.5)	6 (2.4)	1.000
Inotropes or vasopressors	61 (11.5)	34 (12.0)	27 (11.0)	0.824
Length of stay, days (*n* = 503)	7 [5–9]	7 [5–10]	7 [5–9]	0.141
Mortality, whole cohort (*n* = 530; 284 men, 246 women)				
In-hospital death	27 (5.1)	14 (4.9)	13 (5.3)	1.000
In-hospital cardiovascular death	20 (3.8)	12 (4.2)	8 (3.3)	0.720
Death at 12 months from admission	154 (29.1)	86 (30.3)	68 (27.6)	0.568
Postdischarge mortality (*n* = 503; 270 men, 233 women)				
Postdischarge death	127 (25.2)	72 (26.7)	55 (23.6)	0.493
Death by 6 months from admission (hospital survivors)	68 (13.5)	37 (13.7)	31 (13.3)	1.000

Values are *n* (%) or median [interquartile range]. Mortality is reported for the whole cohort and, separately, for the 503 patients discharged alive. In-hospital course is reported in the whole cohort, except length of stay, which is reported for patients discharged alive.

### 3.4 Prognostic model and effect modification by sex

In the multivariable model of postdischarge all-cause mortality (*n* = 494 with complete data, 124 deaths; apparent C-index 0.709, optimism-corrected 0.686, 95% CI 0.644–0.727), log NT-proBNP (HR 1.86 per log unit, 95% CI 1.45–2.38), frailty (HR 1.96, 95% CI 1.09–3.51), and preserved EF (HR 1.71, 95% CI 1.13–2.60) were associated with death after adjustment, whereas age, eGFR, hemoglobin, sodium, and WRF class were not (Table 5). Nine of the 503 patients discharged alive were excluded for missing NT-proBNP; 3 of them died, and hence, the model analyzed 124 of the 127 postdischarge deaths. The excluded and included patients were of similar age (median 80 years in both groups) and sex distribution (three of the nine excluded were men).

**Table 5:** Multivariable cox models for postdischarge all-cause mortality, overall and within each sex.

Covariate	Overall (*n* = 494, 124 deaths)	Men (*n* = 267, 71 deaths)	Women (*n* = 227, 53 deaths)	*p* for interaction
Male sex	1.51 (1.03–2.22)	—	—	—
Age (per 10 years)	1.18 (0.90–1.55)	1.03 (0.73–1.46)	1.43 (0.90–2.26)	0.137
Frailty (CFS ≥ 5)	1.96 (1.09–3.51)	1.89 (0.94–3.80)	2.77 (0.88–8.75)	0.314
Log NT-proBNP	1.86 (1.45–2.38)	1.82 (1.29–2.56)	2.10 (1.42–3.10)	0.204
Baseline eGFR (per 10 mL/min/1.73 m^2^)	0.97 (0.87–1.08)	1.01 (0.88–1.16)	0.93 (0.78–1.11)	0.317
Hemoglobin (per g/dL)	0.99 (0.88–1.11)	0.94 (0.81–1.09)	1.08 (0.90–1.30)	0.425
Sodium (per mEq/L)	1.00 (0.95–1.04)	1.00 (0.94–1.06)	0.99 (0.92–1.06)	0.939
WRF class (per grade)	1.04 (0.88–1.23)	1.08 (0.89–1.31)	0.96 (0.71–1.30)	0.381
LVEF ≥50%	1.71 (1.13–2.60)	2.21 (1.29–3.79)	1.14 (0.61–2.12)	0.060
C-index	0.709	0.716	0.727	—

CFS, clinical frailty scale; eGFR, estimated glomerular filtration rate; LVEF,  left ventricular ejection fraction; NT-proBNP,  N-terminal pro–B-type natriuretic peptide; WRF, worsening renal function.

Hazard ratios with 95% confidence intervals; time zero is discharge. *p* for interaction is the Wald test of a sex-by-covariate interaction term in the combined model. Sex-stratified estimates are descriptive; with eight covariates for 71 and 53 events, they are imprecise. C-indices are apparent values; optimism-corrected estimates with bootstrap confidence intervals are given in the text.

Male sex carried an HR of 1.51 (95% CI 1.03–2.22) in this model. This estimate was not robust to model specification. In the same 494 complete cases, the association was absent without adjustment (HR 1.16, 95% CI 0.81–1.66) and in a model adjusted for age alone (HR 1.30, 95% CI 0.91–1.86). Removing one covariate at a time from the full model identified the LVEF category as the only variable responsible: without it, the HR for male sex fell to 1.28 (95% CI 0.89–1.84, *p* = 0.175), whereas removing any other single covariate left it essentially unchanged (range 1.47–1.53, all *p* < 0.05) (Supplementary Table 1).

None of the eight covariates showed statistically significant effect modification by sex (all *p* for interaction ≥0.06) (Table 5, Figure 2). Fitted separately within each sex, the model gave similar optimism-corrected discrimination in men (*n* = 267, 71 deaths; apparent C-index 0.716, corrected 0.683, 95% CI 0.629–0.736) and women (*n* = 227, 53 deaths; apparent 0.727, corrected 0.687, 95% CI 0.632–0.749), with the small apparent advantage in women removed after correction for optimism. These stratified models contain eight covariates for 71 and 53 events, respectively, and are therefore imprecise; several CIs are wide, and the analysis cannot exclude clinically important heterogeneity, particularly for the LVEF category, for which the interaction was the closest to conventional significance (men 2.21, women 1.14, *p* for interaction 0.060).

**Figure 2:**
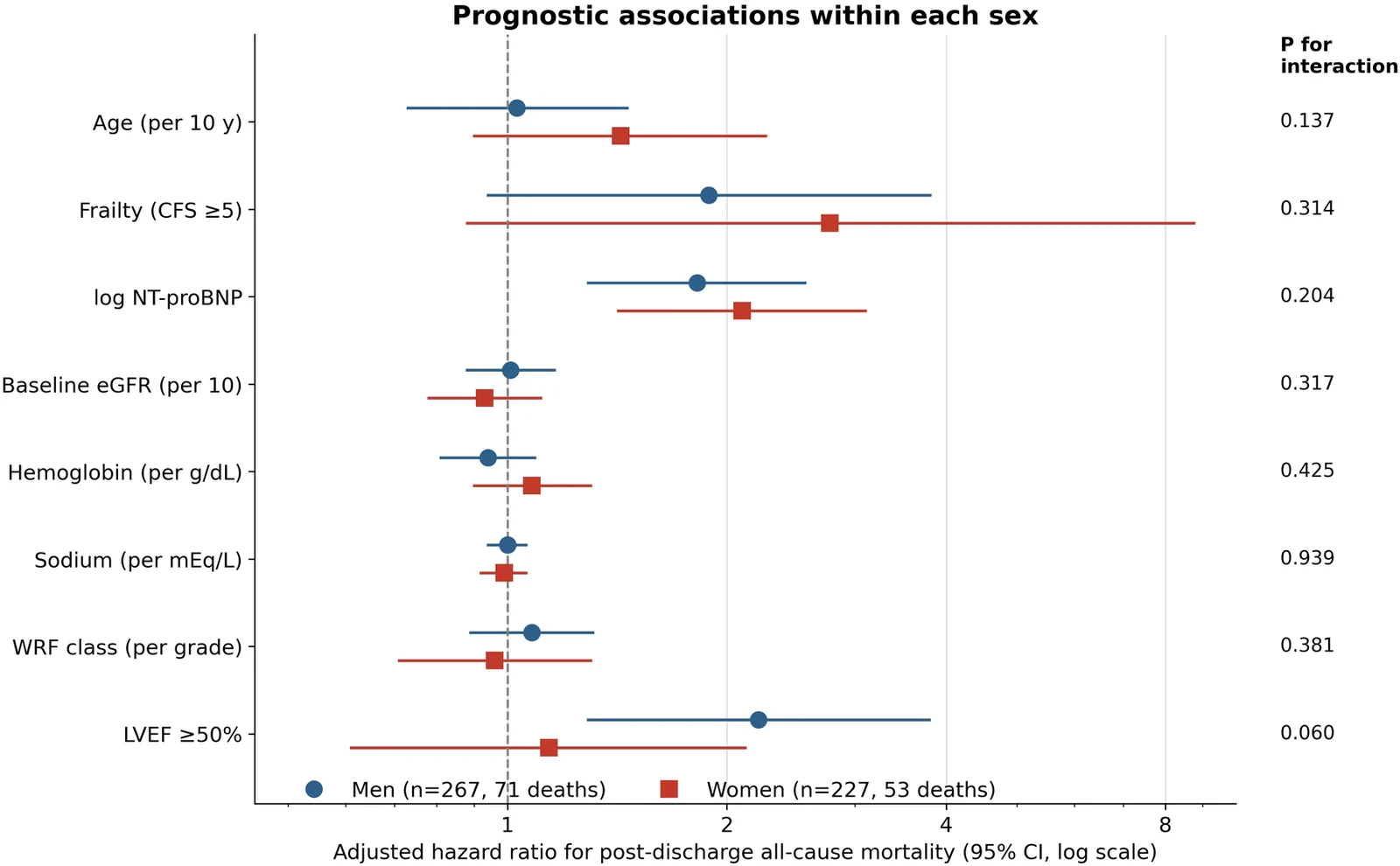
Adjusted hazard ratios for postdischarge all-cause mortality within each sex. The multivariable model was fitted separately in men (*n* = 267, 71 deaths) and women (*n* = 227, 53 deaths); the *p*-value for interaction, from a sex-by-covariate term in the combined model, is shown on the right. The horizontal axis is on a logarithmic scale. These stratified estimates are descriptive and imprecise, with eight covariates for 71 and 53 events, respectively. CFS, clinical frailty scale; eGFR, estimated glomerular filtration rate; LVEF, left ventricular ejection fraction; NT-proBNP, N-terminal pro–B-type natriuretic peptide; WRF, worsening renal function.

The proportional-hazards assumption was met for seven of the nine covariates; hemoglobin showed evidence of a time-varying effect (Schoenfeld *p* = 0.026) and log NT-proBNP was borderline (*p* = 0.059), while all others had *p* ≥ 0.17. The linear form assumed for each continuous covariate was supported by likelihood-ratio tests against restricted cubic splines (age *p* = 0.28, eGFR *p* = 0.58, hemoglobin *p* = 0.44, sodium *p* = 0.44, log NT-proBNP *p* = 0.97). Two sensitivity analyses addressed the hemoglobin violation. Omitting hemoglobin left the model essentially unchanged (male sex HR 1.51, 95% CI 1.03–2.22; LVEF ≥50% HR 1.72), as did stratifying the model on the tertiles of hemoglobin (male sex HR 1.54, 95% CI 1.05–2.25; LVEF ≥50% HR 1.75, *p* = 0.009). No interaction conclusion changed in either analysis.

### 3.5 Metabolic and inflammatory markers

Each of 19 metabolic, inflammatory, and renal markers was added in turn to the core model together with a marker-by-sex interaction term. Eighteen showed no evidence of effect modification (*p* for interaction 0.08–0.95). Uric acid was the exception: each 1 mg/dL increment was associated with higher postdischarge mortality rates in women (HR 1.15, 95% CI 1.00–1.33) than in men (HR 0.91, 95% CI 0.80–1.03), interaction *p* = 0.008 (Supplementary Table 2).

This estimate was stable. The interaction persisted when the model was reduced to age alone (*p* = 0.005), when the eGFR was omitted (*p* = 0.009), and after further adjustment for diabetes (*p* = 0.008), diuretic dose and MRA use (*p* = 0.012), CRP (*p* = 0.006), hemoglobin and albumin (*p* = 0.016), and WRF class (*p* = 0.008). It remained after the 15 patients with uric acid above 12 mg/dL (*p* = 0.022) were excluded, and no single observation altered the interaction coefficient by more than 13%. Uric acid was measured in 414 of the 530 patients (78%), with equal frequency in men and women (77.8% vs. 78.5%, *p* = 0.94), and no difference in mortality rates between those measured and not measured was observed (*p* = 0.85) (Supplementary Table 3). Of these 414, 393 were discharged alive and 386 also had complete data for the covariates of the core model; therefore, the primary uric acid model includes 386 patients, the reduction from 414 reflecting in-hospital death and missing NT-proBNP rather than any additional missingness of uric acid itself.

Two features temper this finding. It does not survive correction for the 19 comparisons performed (Bonferroni threshold *p* < 0.0026), and it was not seen when the endpoint was the composite of death, HF rehospitalization, or RRT (interaction *p* = 0.094). The linear form assumed for uric acid was, however, supported by a likelihood-ratio test against a restricted cubic spline (*p* = 0.43).

One additional nominal interaction, for CKD as a binary variable (*p* = 0.079), had a biologically implausible direction in men and is attributed to collinearity with the eGFR, which was already present in the model; therefore, it is not interpreted in detail.

## 4 Discussion

In this consecutive cohort of patients hospitalized for AHF, there were substantial differences between the sexes in terms of what they presented and hardly anything at all in terms of their outcomes. The phenotypic separation was the most robust finding of this study: large, consistent across clinical, echocardiographic, and biochemical domains, and evident in variables measured in every patient. Compared with men, women were older, frailer, more frequently presented with AF, more often had preserved EF and more advanced CKD, had a higher E/e′ ratio within preserved EF, and carried a distinct metabolic signature of higher cholesterol fractions and markedly lower ferritin levels, while ischemic etiology dominated in men. Yet, unadjusted in-hospital and postdischarge all-cause mortality rates were similar between the sexes, with the excess rates in men after full adjustment depending on the inclusion of the LVEF category. No statistically significant sex differences were observed in the renal outcomes examined, and we found no statistically robust evidence that the markers used to estimate risk carried different weights in the two sexes, although several sex-specific estimates had limited accuracy.

This pattern is consistent with what has been described in larger registries and trials, in which women present later with more preserved systolic function and a heavier burden of frailty and AF, while ischemic disease dominates in men (1, 3). The same dissociation between phenotype and prognosis was reported in the HELP-HF registry of 1,149 patients with markers of advanced HF, in which men and women differed in terms of clinical and echocardiographic profile yet had virtually identical one-year rates of death or HF hospitalization (43.2% vs. 43.1%; adjusted HR 1.03, 95% CI 0.85–1.27) (16). The predominance of preserved EF among women also places most of them in the phenotype that European registries have shown to be the oldest and most comorbid, and the one in which mortality is driven least by EF itself (17, 18). The predictors that did carry weight—natriuretic peptide and frailty—are those with the most established prognostic role in this population (19–21). That in-hospital mortality was identical despite these opposing risk profiles suggests that the older age and greater frailty of women were offset by the more frequent ischemic etiology and lower EF of men, although the small number of in-hospital deaths precludes formal testing.

The echocardiographic data add one observation that is not simply a restatement of the difference in EF. The more frequent LV dilation in men is expected from their lower EF and ischemic substrate, and it persisted within the LVEF <50% group. The higher E/e′ ratio in women, by contrast, was confined to patients with LVEF ≥50%, where it was higher in women than in men with the same phenotype, and the higher PASP in women disappeared within each LVEF group. Within preserved EF, women therefore showed a more marked Doppler evidence of diastolic dysfunction, while the difference in pulmonary pressures appears to reflect the different phenotype mix. RV size and function, in contrast, were similar in the two sexes, and therefore, the higher prevalence of moderate or severe TR in women was not accompanied by higher RV dysfunction. Echocardiography was performed once, during the congested phase, and hence, these values describe the decompensated state rather than a stable cardiac structure.

Two additional differences bear on interpretation. First, coronary anatomy had not been assessed in almost half of the women, twice the proportion in men, and therefore, the lower prevalence of ischemic etiology in women partly reflects less frequent coronary evaluation and may underestimate ischemic disease in this group. The lower admission troponin in women is consistent with a smaller ischemic contribution but does not resolve this uncertainty. Second, women were discharged less often on SGLT2i inhibitors or a statin, although beta-blocker, MRA, and RAS inhibitor prescriptions were similar. Within each LVEF group, however, SGLT2i prescription did not differ between the sexes (51.6% vs. 56.4% with LVEF ≥50% and 71.8% vs. 76.9% with LVEF <50%), and therefore, the overall difference reflects the lower prescription in preserved EF, which was the category most women belonged, rather than a sex-specific disparity. Given that SGLT2i inhibitors are now recommended across the EF spectrum (11), this lower uptake in preserved EF represents a potentially modifiable gap in care.

What our data add is that the divergence between the sexes extends into the metabolic and inflammatory domains without a robust difference in prognosis. Read with the caveat above, the difference appears to lie more in the profile that patients bring to hospital than in how that profile should be read, and these data do not support sex-specific prognostic thresholds for the markers examined. Optimism-corrected discrimination was almost identical in men and women, which argues against the concern that models derived in male-predominant cohorts perform substantially worse in women, although equivalence cannot be established from a cohort of this size.

The lipid findings deserve comment, because higher total and LDL cholesterol in women might appear paradoxical in a population that was also frailer. In advanced HF, low cholesterol is a recognized marker of catabolic state rather than of low cardiovascular risk (22), and the higher values in women may partly reflect differences in nutritional, inflammatory or HF phenotype, although cachexia was not directly assessed and neither body mass index nor weight change value was available. Consistent with this, neither total nor LDL cholesterol predicted mortality in either sex.

Low ferritin was the most clinically relevant difference that we observed. Iron deficiency is common in HF and is a treatable target (23, 24), and therefore, a difference of this magnitude is potentially important. It must, however, be interpreted with care: transferrin saturation was not measured, and therefore, functional iron deficiency could not be identified, and this finding does not establish a higher prevalence of guideline-defined iron deficiency in women. Ferritin also behaves as an acute-phase reactant in hospitalized patients. This observation indicates where complete iron indices would be informative rather than what proportion of women require treatment.

The one signal of effect modification concerned uric acid, associated with postdischarge mortality in women but not in men. This association was consistent across model specifications and was not driven by extreme values or any single patient. Uric acid has an established prognostic association in chronic HF (25), urate handling differs by sex, and uric acid thresholds for cardiovascular mortality lower than the conventional sex-specific definitions of hyperuricemia have been identified (26), and hence, the finding is biologically plausible. Should this association prove reproducible, two mechanisms might explain why the same uric acid concentration carried more prognostic weight in women. First, estrogen-mediated uricosuria keeps uric acid lower in women, so that a given concentration represents a larger deviation from their physiological range, and hyperuricemia in women has been described as a marker of escape from estrogen-mediated cardiovascular protection (27); consistent with this, women in the present cohort had similar or lower uric acid levels than men at comparable eGFR (data not available). Second, elevated uric acid levels in HF partly reflect an upregulation of xanthine oxidase, a major source of oxidative stress linked to endothelial and diastolic dysfunction (27, 28), a pathway plausibly more relevant in women, who predominantly had preserved EF and a higher E/e′ ratio. Renal function did not account for the interaction, which persisted with and without the eGFR in the model and after adjustment for diuretic dose and MRA use. However, we tread with caution: it was one of 19 comparisons and did not survive correction for multiplicity, it was absent for the composite endpoint, and it was graded rather than threshold-dependent, and hence, no cut point can be proposed. It is hypothesis-generating and requires confirmation in an independent cohort.

Two coefficients in the adjusted model require exercising the same caution. Crude postdischarge mortality was almost identical in men and women, and the apparent excess in men emerged only in the fully adjusted model, however disappearing when the EF category was removed. In the fully adjusted model, LVEF ≥50% was associated with higher postdischarge mortality, a direction that contrasts both with the crude comparison in the present cohort and with the unadjusted analysis of LVEF phenotype previously reported from this registry (7), and this reflects the older, frailer, and more comorbid preserved-LVEF group. It is best understood as a model-dependent suppression effect and should not be interpreted causally or described as an independent predictor. Because EF phenotype is strongly patterned by sex and drives the adjusted sex coefficient, both estimates may reflect confounding, suppression, or a different estimand depending on specification, and both are reported as features of the adjusted model rather than as independent effects.

### 4.1 Limitations

This was a single-center study in an elderly Mediterranean population, and the findings may not transfer to younger or more ethnically diverse cohorts. Body weight and height were not systematically recorded, and therefore, body mass index could not be derived and the obesity-related phenotype could not be characterized. Sex was obtained from the medical record and no information on gender identity was collected; therefore, the analysis addresses only sex and not gender. Uric acid, ferritin, and albuminuria levels were not available in all patients, although missingness was similar between the sexes; transferrin saturation was not measured, limiting interpretation of the ferritin findings. Baseline creatinine was selected clinically as the most representative stable preadmission value rather than by an automated rule, which may introduce measurement error into baseline eGFR and KDIGO staging. Coronary anatomy had not been assessed in a third of patients, more often in women, and hence, ischemic etiology may be underascertained; discharge therapy was not included in the prognostic model.

Several statistical limitations apply. With 71 deaths in men and 53 in women, the sex-stratified models contain eight covariates for fewer than nine events per variable and are consequently imprecise and at risk of overfitting; the interaction analyses have limited power, and the absence of effect modification should be read as absence of evidence rather than as evidence of equivalence. Hemoglobin showed evidence of a time-varying effect, and therefore, its HR should be interpreted as an average over follow-up; sensitivity analyses omitting hemoglobin and stratifying on hemoglobin tertiles left the sex coefficient and the interaction conclusions unchanged. Follow-up ended 12 months after admission rather than 12 months after discharge, and hence, the postdischarge observation window varied with the length of stay; the median length of stay was identical in both sexes, making differential follow-up an unlikely source of bias, but this was not formally adjusted for. Finally, the 19 interaction tests were only exploratory and not adjusted for multiplicity, and the analysis was *post hoc* rather than prespecified. Most importantly, the principal findings—the sex-related phenotypic differences and the uric acid interaction—derive from a single center and have not been externally validated; they should be regarded as hypothesis-generating and require confirmation in independent, multicenter cohorts before any clinical inference is made.

### 4.2 Conclusions

Men and women hospitalized for AHF represent clearly distinct clinical and metabolic phenotypes. In this study, it was found that women were older and frailer than men, predominantly had preserved EF and more advanced CKD, and carried a different metabolic signature of higher cholesterol fractions and substantially lower ferritin levels then men; in contrast, men were younger, predominantly ischemic, and less frail. These are large and consistent differences, measured in every patient, and they define two populations that meet in the same ward for the same syndrome. Differences in coronary evaluation, diastolic function, and discharge therapy accompany this phenotypic separation and should be considered as potential confounders when metabolic and inflammatory markers are compared between the sexes. Unadjusted mortality, in the hospital and after discharge, was nevertheless similar in both men and women; the adjusted association of male sex with postdischarge mortality was sensitive to the inclusion of the LVEF category and therefore, should be interpreted cautiously. No statistically robust evidence of sex-related effect modification was identified for the routinely measured prognostic markers examined. Several sex-specific estimates had limited accuracy, and this finding represents the absence of evidence rather than evidence of equivalence. A ferritin level below 100 ng/mL was considerably more frequent in women than in men and warrants further evaluation using complete iron indices. The nominal interaction between sex and uric acid was the single exception among the 19 markers examined, did not withstand correction for multiple testing, and should be considered hypothesis-generating.

## Data Availability

The data are available from the corresponding author on reasonable request, subject to institutional and ethics-committee restrictions on patient data.
