# Supplementary material for: Sex differences in clinical phenotypes and prognostic associations in acute heart failure
Source: Front Cardiovasc Med. 2026 Oct 28;13:2011890. doi: 10.3389/fcvm.2026.2011890 (PMC13752040; doi:10.3389/fcvm.2026.2011890)
Supplement: Supplementary file 1 [file Table1.docx]

**Supplementary Material**

*Sex Differences in Clinical Phenotypes and Prognostic Associations in Acute Heart Failure*

**Supplementary Table S1. Hazard ratio for male sex across specifications of the primary model**

| **Model** | **n** | **HR for male sex (95% CI)** | **p** |
| --- | --- | --- | --- |
| Unadjusted | 494 | 1.16 (0.81–1.66) | 0.410 |
| Age only | 494 | 1.30 (0.91–1.86) | 0.149 |
| Full model (all nine covariates) | 494 | 1.51 (1.03–2.22) | 0.034 |
| Full model without age | 494 | 1.53 (1.04–2.25) | 0.029 |
| Full model without frailty | 494 | 1.47 (1.01–2.15) | 0.046 |
| Full model without log NT-proBNP | 494 | 1.49 (1.02–2.17) | 0.041 |
| Full model without eGFR | 494 | 1.51 (1.03–2.22) | 0.034 |
| Full model without hemoglobin | 494 | 1.51 (1.03–2.22) | 0.034 |
| Full model without sodium | 494 | 1.51 (1.03–2.22) | 0.034 |
| Full model without WRF class | 494 | 1.52 (1.04–2.23) | 0.032 |
| Full model without LVEF category | 494 | 1.28 (0.89–1.84) | 0.175 |

Post-discharge all-cause mortality in the 503 patients discharged alive, with time zero at discharge. All models are fitted in the same 494 patients with complete data (n=494; 124 deaths [71 men, 53 women]), so the specifications are directly comparable. The full model comprises sex, age, frailty, log NT-proBNP, baseline eGFR, hemoglobin, sodium, worsening renal function class and LVEF category, and is identical to the model in Table 5; each subsequent row removes one covariate at a time. Removing LVEF category was the only change that abolished the association between male sex and post-discharge mortality; removing any other single covariate left the hazard ratio between 1.47 and 1.53. Because preserved ejection fraction was considerably more frequent in women (65.9% versus 35.9%) and was itself associated with higher post-discharge mortality in the adjusted model, the sex coefficient is sensitive to how the ejection fraction phenotype is modeled and is not interpreted as an independent effect of sex. CI = confidence interval; eGFR = estimated glomerular filtration rate; HR = hazard ratio; LVEF = left ventricular ejection fraction; NT-proBNP = N-terminal pro–B-type natriuretic peptide; WRF = worsening renal function.

**Supplementary Table S2. Sex interaction for metabolic, inflammatory and renal markers**

| **Marker** | **Unit** | **Men, HR (95% CI)** | **Women, HR (95% CI)** | **p for interaction** | **n** |
| --- | --- | --- | --- | --- | --- |
| Uric acid | per mg/dL | 0.91 (0.80–1.03) | 1.15 (1.00–1.33) | 0.008 | 386 |
| Chronic kidney disease | yes vs no | 0.32 (0.12–0.82) | 1.43 (0.50–4.09) | 0.079 | 494 |
| Low ferritin | yes vs no | 0.56 (0.34–0.91) | 1.06 (0.56–1.99) | 0.141 | 475 |
| White cell count | per SD | 0.98 (0.74–1.30) | 0.78 (0.58–1.06) | 0.220 | 494 |
| LDL cholesterol | per SD | 0.87 (0.65–1.15) | 0.75 (0.55–1.02) | 0.357 | 493 |
| Platelet-to-lymphocyte ratio | per SD | 1.14 (0.93–1.39) | 0.97 (0.75–1.25) | 0.360 | 494 |
| Worsening renal function class | per grade | 1.09 (0.90–1.32) | 0.96 (0.71–1.29) | 0.376 | 494 |
| Admission glucose | per log unit | 1.52 (0.75–3.10) | 1.01 (0.43–2.35) | 0.386 | 494 |
| Triglycerides | per SD | 1.16 (0.90–1.49) | 0.99 (0.70–1.40) | 0.434 | 493 |
| Albumin | per g/dL | 0.64 (0.32–1.25) | 0.50 (0.25–0.97) | 0.530 | 489 |
| Total cholesterol | per SD | 0.85 (0.64–1.13) | 0.76 (0.57–1.03) | 0.531 | 492 |
| Diabetes mellitus | yes vs no | 1.06 (0.65–1.73) | 1.41 (0.80–2.49) | 0.587 | 494 |
| Neutrophil-to-lymphocyte ratio | per log unit | 1.10 (0.78–1.55) | 0.98 (0.66–1.47) | 0.589 | 494 |
| Red cell distribution width | per % | 1.06 (0.99–1.13) | 1.09 (0.98–1.22) | 0.639 | 494 |
| HbA1c | per % | 1.12 (0.94–1.34) | 1.08 (0.88–1.34) | 0.700 | 486 |
| Monocyte-to-lymphocyte ratio | per log unit | 0.92 (0.63–1.35) | 0.85 (0.54–1.35) | 0.735 | 494 |
| C-reactive protein | per log unit | 1.12 (0.89–1.41) | 1.05 (0.78–1.41) | 0.772 | 494 |
| HDL cholesterol | per SD | 0.83 (0.63–1.09) | 0.82 (0.61–1.09) | 0.905 | 492 |
| Ferritin | per log unit | 1.08 (0.83–1.39) | 1.06 (0.81–1.39) | 0.948 | 475 |

Each marker was added in turn to a core model comprising age, frailty, log NT-proBNP, estimated glomerular filtration rate and left ventricular ejection fraction category, together with both main effects (sex and the marker) and a marker-by-sex interaction term; the p value for interaction is the Wald test of that term in the combined model; the endpoint was post-discharge all-cause mortality with time zero at discharge. Within-sex hazard ratios are from the same model fitted separately in men and women. Markers are ordered by the p value for interaction. Numbers refer to patients discharged alive with complete data for the core model and the marker concerned. The nineteen markers tested are those listed in this table; thyroid-stimulating hormone and magnesium were measured and described in Table 3 of the main text but were not included in the interaction screen. The Bonferroni-corrected threshold is p<0.0026, which none of the interactions meets. The nominal interaction for chronic kidney disease has a biologically implausible direction in men and is attributed to collinearity with estimated glomerular filtration rate, which was already in the model; it is not interpreted. CI = confidence interval; HbA1c = glycated hemoglobin; HR = hazard ratio; SD = standard deviation.

**Supplementary Table S3. Sensitivity analyses of the uric acid–sex interaction**

| **Analysis** | **Men, HR per mg/dL (95% CI)** | **Women, HR per mg/dL (95% CI)** | **p for interaction** | **n** |
| --- | --- | --- | --- | --- |
| Primary model | 0.91 (0.80–1.03) | 1.15 (1.00–1.33) | 0.008 | 386 |
| **Alternative adjustment sets** |  |  |  |  |
| Age only | 0.94 (0.84–1.06) | 1.22 (1.07–1.39) | 0.005 | 393 |
| Without estimated glomerular filtration rate | 0.91 (0.80–1.02) | 1.16 (1.01–1.34) | 0.009 | 386 |
| Plus diabetes mellitus | 0.91 (0.80–1.03) | 1.15 (0.99–1.32) | 0.008 | 386 |
| Plus furosemide dose and MRA use | 0.88 (0.78–1.01) | 1.12 (0.96–1.29) | 0.012 | 386 |
| Plus log C-reactive protein | 0.90 (0.79–1.03) | 1.16 (1.00–1.34) | 0.006 | 386 |
| Plus hemoglobin and albumin | 0.92 (0.81–1.05) | 1.15 (0.99–1.33) | 0.016 | 386 |
| Plus worsening renal function class | 0.91 (0.80–1.03) | 1.15 (0.99–1.33) | 0.008 | 386 |
| **Influence and distribution** |  |  |  |  |
| Excluding uric acid >12 mg/dL | 0.87 (0.76–1.00) | 1.13 (0.94–1.35) | 0.022 | 371 |
| **Alternative endpoint** |  |  |  |  |
| Composite of death, HF rehospitalization or RRT | 0.95 (0.87–1.04) | 1.04 (0.94–1.15) | 0.094 | 386 |

All models are Cox proportional-hazards models for post-discharge all-cause mortality with time zero at discharge, unless otherwise stated. The primary model comprised age, frailty, log NT-proBNP, estimated glomerular filtration rate and left ventricular ejection fraction category. In addition to the analyses tabulated, a likelihood-ratio test of the interaction term gave χ²=7.04 (p=0.008), and leave-one-out analysis showed that no single observation altered the interaction coefficient by more than 13%. These analyses concern this single marker and do not correct for the nineteen markers screened. Uric acid was measured in 414 of 530 patients (78.1%); of these, 393 were discharged alive and 386 also had complete covariate data, which is the sample of the primary model. Measurement was similar in men and women (77.8% versus 78.5%, p=0.943), and one-year mortality did not differ between patients in whom uric acid was and was not measured (28.7% versus 30.2%, p=0.854). CI = confidence interval; HF = heart failure; HR = hazard ratio; MRA = mineralocorticoid receptor antagonist; RRT = renal replacement therapy.
